# A Critical Look at Biomedical Journals’ Policies on Animal Research by Use of a Novel Tool: The EXEMPLAR Scale

**DOI:** 10.3390/ani5020315

**Published:** 2015-04-30

**Authors:** Ana Raquel Martins, Nuno Henrique Franco

**Affiliations:** 1 Faculty of Sciences, University of Porto, Rua do Campo Alegre S/N, 4169-007 Porto, Portugal; E-Mail: up201101588@fc.up.pt; 2 IBMC—Instituto de Biologia Molecular e Celular, University of Porto, Rua do Campo Alegre 823, 4150-180 Porto, Portugal

**Keywords:** animal research, animal ethics, animal welfare, editorial policies, EXEMPLAR scale

## Abstract

**Simple Summary:**

Biomedical journals have the responsibility to promote humane research. To gauge and evaluate journal policies on animal research, the *EXEMPLAR*—For “Excellence in Mandatory Policies on Animal Research”—scale is presented and applied to evaluate a sample of 170 biomedical journals, providing an overview of the current landscape of editorial policies on the ethical treatment of animals.

**Abstract:**

Animal research is not only regulated by legislation but also by self-regulatory mechanisms within the scientific community, which include biomedical journals’ policies on animal use. For editorial policies to meaningfully impact attitudes and practice, they must not only be put into effect by editors and reviewers, but also be set to high standards. We present a novel tool to classify journals’ policies on animal use—the *EXEMPLAR* scale—as well as an analysis by this scale of 170 journals publishing studies on animal models of three human diseases: Amyotrophic Lateral Sclerosis, Type-1 Diabetes and Tuberculosis. Results show a much greater focus of editorial policies on regulatory compliance than on other domains, suggesting a transfer of journals’ responsibilities to scientists, institutions and regulators. Scores were not found to vary with journals’ impact factor, country of origin or antiquity, but were, however, significantly higher for open access journals, which may be a result of their greater exposure and consequent higher public scrutiny.

## 1. Introduction

Animal experimentation has, for centuries, not only been a cornerstone of scientifically-based biomedical research, but also the subject of heated academic and public debate [1]. Given the number and complexity of the arguments at play in this discussion, views on the moral acceptability of animal experiments are diverse, most of which fall in between the diametrically opposed views of those uncompromisingly against this practice and the more determined advocates of animal experimentation. Such diversity results from the interplay between such factors—among others—as the suffering and discomfort endured by animals [2,3,4], the purpose and validity of research [4,5,6,7,8], which species are used [6,9,10,11] or the degree of confidence in scientists and regulators [3,12,13], as well as the different positions on the weighting that should be given to each factor.

In spite of this multitude of positions, in western societies, the majority of the public tends to follow a moderate, mainstream view on the subject, according to which animal experimentation is seen as a legitimate and ethically acceptable scientific activity, provided it is guided by a scientifically sound and medically relevant objective, and that animal welfare is taken into consideration [8,13]. This “conditional approval” view is in line with the tenet of the Three Rs [14]—for the Replacement, Reduction and Refinement of animal use in science—widely accepted as a scientifically sound approach to help address the ethical dilemma put forth by animal research [8,15], as well as an overarching principle in current European legislation regulating animal use for scientific purposes [16].

The mandate of scientists to work with animals requires a continually renewed relationship of trust with society, whose views and expectations, on the other hand, legislators also take into account when establishing the legal framework within which scientists must work. This tacit “social license” granted to scientists warrants public trust in the relevance, competency, reliability, ethicality and transparency of animal experiments, which goes beyond the mere assumption of compliance with laws and regulations [17,18]. From this interplay results a tightly regulated environment for animal research, by both ‘external’ regulatory frameworks (such as legislation [16,19]) and self-regulatory initiatives from within the scientific community itself. These include peer review [20], training [21,22], the issuing of publication guidelines (such as ARRIVE [23] or the *Gold Standard Publication Checklist* [24]) or other scientific community-driven initiatives, such as the Basel Declaration for transparency in animal research [25]. Biomedical journals’ policies also have great potential for improving principles and practice in animal research, since the motivation—and often the pressure—to publish may serve as an incentive for scientists to comply with journals’ demands [26,27] on such issues as quality of research and reporting, or the ethical treatment of animals, particularly for high-impact journals.

Given the potential of scientific journals for promoting best practice in animal research, evaluating their level of concern for animal welfare and the quality of research becomes of the essence. In 2009, RSPCA’s Nicola Osborne *et al.* [28] proposed a 12-item (each awarding one point) classifying scheme for scoring biomedical journals’ policies on animal welfare and the Three Rs, and presented results of a review of 236 biomedical journals’ policies. They found that in 35% of their sample, animal use was not even contemplated in the guidelines for authors or anywhere else, in 18% animals were mentioned in some way but no perceptible guidelines were provided and the remaining scored rather poorly, with 37% scoring three or fewer points (out of 12).

**Table 1:** The Excellence in Editorial Mandatory Policies for Animal Research (EXEMPLAR) scale.

EXEMPLAR Scale		Score
**A—Reporting of regulatory compliance**		
Authors must attest of prior ethical approval of animal studies, or an analogous project evaluation process involving harm-benefit appraisal, (e.g., by providing documental evidence or ethics review board process reference) and include a statement of compliance with relevant legislation and national, international or institutional guidelines on animal care and use.		**5**
Authors must declare:	Ethical approval of studies or analogous evaluation process by competent authority, institutional animal care and use committee, animal welfare body or equivalent.	2
	Compliance with relevant national, international or institutional guidelines on animal care and use.	1
	Compliance with relevant legislation on the use of animals.	1
**B—Quality of research and reporting of results**		
Journal refers authors to relevant guidelines on the quality of reporting of animal studies (e.g., ARRIVE guidelines, Gold Standard Publication Checklist, or other)		**5**
Authors must include information regarding:	Animals (e.g., sex, age, genotype, background, supplier, acclimatization period, *etc.*)	1
	Experimental conditions (e.g., housing, lighting, temperature, feeding regime, environmental enrichment, *etc.*)	1
	Experimental design and statistics	1
	Experimental procedures and outcomes	1
**C—Animal Welfare and ethics**		
Journal demands that the methods described are coherent with best principles and practice on the ethical treatment of animals in research, e.g., by demanding strict compliance with relevant guidelines on animal care and use, the 3Rs for *replacement*, *reduction* and *refinement* or the journal's own policies.		**5**
Authors must:	state the rationale for choosing the animal model(s) used	1
	report the impact of the experiment on animal health and wellbeing	1
	describe any measures to minimize harm (e.g., anesthetics, analgesics, humane endpoints) and/or improve the wellbeing of animals (e.g., husbandry adaptations for more vulnerable animals)	1
	justify the necessity of any unrelieved pain, suffering or distress inflicted	1
**D—Criteria for the exclusion of papers**		
Meeting journal standards on animal ethics care and welfare is indispensable for manuscript acceptance and/or publication. Studies raising serious concerns over animal welfare, or presenting significant discrepancies between the approved protocol and methods described may be reported to the institution or committee responsible for ethical approval.		**5**
Studies raising serious ethical concerns (e.g., serious neglect regarding animal welfare or unjustifiable suffering considering the value of the experiment) may be rejected by editors or reviewers.		3
Journal states specific procedure(s) that will not be accepted for publication (e.g., use of muscle relaxants or paralytic drugs alone for surgery, severe lesion/trauma without anesthesia, or death as an endpoint)		1

The present study presents the “EXEMPLAR”—an acronym for “Excellence in Editorial Mandatory Policies for Animal Research”—Scale (Table 1) as a novel approach and tool to classify and evaluate scientific journals’ editorial policies on animal use. The EXEMPLAR Scale aims to (a) gauge the consideration (or lack thereof) given by journals to the most relevant aspects of animal use in the life sciences; (b) present a novel way for scoring editorial policies and (c) propose a set of ideal standards for three key aspects in which journal policies can have an instrumental role in promoting best practice: regulatory compliance, quality of reporting and animal welfare and ethics; along with a fourth category for the enforcement of said policies.

This paper also presents an overview of the current landscape of biomedical journals’ policies, by reviewing and classifying by the EXEMPLAR a sample of 170 journals publishing animal studies within three fields of biomedical research: Amyotrophic Lateral Sclerosis (ALS), Type 1 Diabetes (T1D) and Tuberculosis (TB). These fields were selected for being the subject of ongoing systematic reviews in our lab—which will later allow for a comparison between the principles reflected in explicit policies and the actual practice patent in the articles published by the journals in our sample—which in turn were selected for representing areas in which animal experimentation is recurrent and widespread.

## 2. Experimental Section

### 2.1. The Exemplar Scale

The EXEMPLAR scale (Table 1) was developed to classify scientific journals’ editorial policies on animal studies, scoring them from zero to 20 points. The scale is divided into four categories, each of them regarding a relevant aspect of journal policies on animal research. The maximum score for each category is five points, granted to journals that uphold to the scale’s gold-standard for that category. For any journal not abiding to the category’s “gold-standard” criterion, up to four points can be attributed if it nevertheless abides to other relevant criteria listed for that category, each deemed a “standard” criterion.

Categories are divided as such: Category A—Regulatory compliance; Category B—Quality of research and reporting of results, Category C—Animal welfare and ethics; Category D—Criteria for exclusion of papers. Both Categories B and C have four standard criteria, each one worth one point. Category A has three standard criteria, with the item “authors must declare ethical approval of studies or analogous project evaluation process (…)” awarding two points. This is based on the assumptions that (a) project approval involves some level of harm-benefit appraisal by a third party—such as the competent authority or an ethics committee—and (b) that the latter is of greater significance for the humane treatment of animals than plain self-reported compliance with laws and legislation. Category D only has two standard criteria, one of which awarding three points to journals which state that papers may be reject upon ethical concerns. This also follows the rationale that some criteria are of greater relevance than others, in this case, the mere listing of procedures the journal is not willing to publish.

For reporting purposes, the overall EXEMPLAR score is presented along with a breakdown of the partial scores for each category, allowing for anyone knowledgeable of the scale to identify in which dimensions a given journal’s policy is stronger or, on the other hand, where further development is due. For example, the hypothetical *EXEMPLAR score = **12**: (A-5; B-5; C-2; D-0)*—or ***12**:(5,5,2,0)*—would suggest that the journal in question follows “gold standard” criteria for both Categories A and B, that it fulfils half the criteria for Category C and that no policies regarding Category D are described.

### 2.2. Journals Search

**Figure 1:**
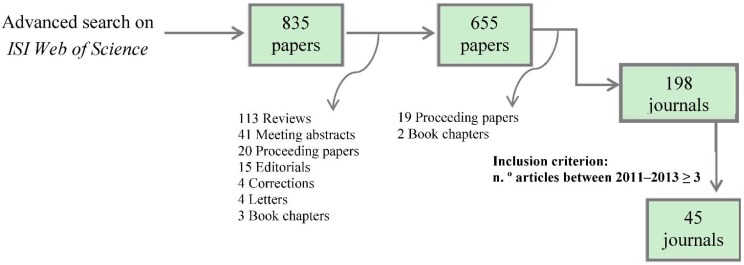
Schematic representation of the search and selection process, with the search for journals publishing studies on Type 1 Diabetes (T1D) being presented as an example. One of the journals from the T1D sample was later excluded because their editorial polices stated that it did not publish studies on animal models, leaving a final sample size of 44 journals.

A list of papers reporting studies on murine models of Type-1 Diabetes (T1D), Tuberculosis (TB) and Amyotrophic Lateral Sclerosis (ALS) published between 2011 and 2013 was retrieved by a ISI Web of Science™ (Core Collection v5.13.1) advanced search. The search was carried out in April 2014 using the queries TS= ((“NOD mouse” OR “NOD mice” OR “non obese diabetic” OR “nonobese diabetic”) AND diabet*); *TS = ((mice OR mouse) AND tuberculosis*); and *TS = ((mice OR mouse) SAME (ALS OR “amyotrophic lateral sclerosis”))*. The results were refined to only include articles in English reporting original research, and afterwards downloaded and archived as an ENDNOTE^®^ database. After being refined, search results were as such: T1D: 655 papers; TB: 1107 papers; ALS: 1114 papers. The criterion for selecting which journals to classify was to only include those that had published three or more papers in each field over the time period selected (Figure 1). This resulted in a sample of 44 journals for T1D (which published 475/655, or 73%, of T1D papers retrieved); 65 journals for TB (which published 999/1107, or 72% of all TB papers retrieved) and 84 journals for ALS (which published 830/1114, or 75%, of all ALS papers retrieved).

### 2.3. Data Collection

Journal policies were collected by finding the “authors guidelines”, “Instructions for authors” sections or equivalent on each journal’s website, and saving them to a database. Whenever a journal required authors to comply with general policies of its publishing house, or subscribed to other guidelines (e.g., by the International Association of Veterinary Editors), these were also considered as a part of the journal’s policies.

General information on each journal—impact factor, publisher, country and scientific category—were collected from the ISI Web of Knowledge Journal Citation Reports^®^, JCR Science Edition 2013, while information regarding the date (year) of the first issue and model of publication—*i.e.*, subscription based *vs.* open access—were retrieved from each journal’s website. Only journals in which all published papers were readily and freely accessible without any embargo period were considered to be “open access”.

All editorial policies were classified according to both the EXEMPLAR Scale (Table 1) and the classification scheme proposed by Osborne *et al.* [28].

### 2.4. Statistical Analysis

Chi-square tests were conducted to assess the relationship between the EXEMPLAR Score (ES) and qualitative variables (model of publication, country, and publisher), while for quantitative variables (impact factor, first year of edition and the score by the Osborne *et al.* scale) a logistic regression analysis was also performed. Linear relationships were assessed by the Pearson test. For some non-parametric statistical tests, the variable “ES” was transformed into a dichotomous variable, ES < eight and ES ≥ eight. A score of eight points was selected as a threshold for a minimally acceptable policy on animal use, since it can be awarded to a journal that complies with half the “standard” criteria defined for each category. Statistical analysis was conducted using SPSS Statistics 22 (IBM, Armonk, NY, USA; version 22.0).

## 3. Results and Discussion

### 3.1. Sample Characterization

**Figure 2:**
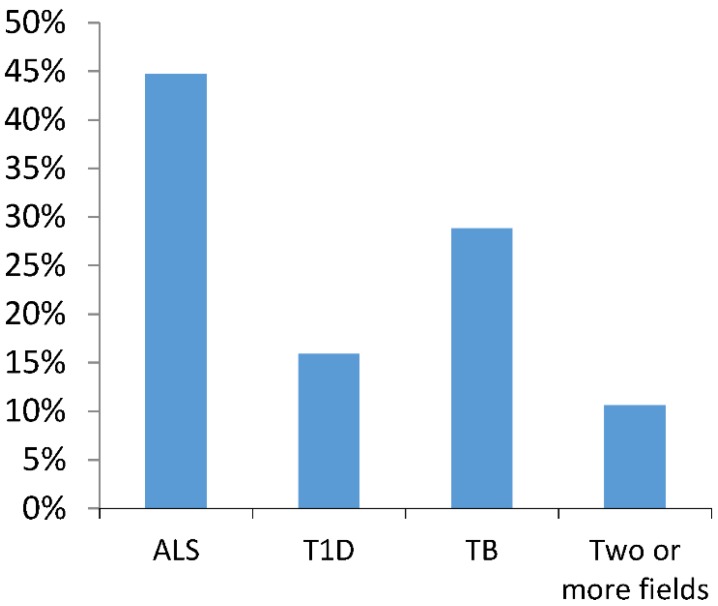
Topics covered by the journals in the sample (of the three selected). Journals publishing studies in more than one of the selected fields (n = 18) included two journals publishing papers on T1D and ALS, one journal publishing on Amyotrophic Lateral Sclerosis (ALS) and Tuberculosis (TB), ten journals publishing on both T1D and TB and five journals publishing papers on all three fields, between 2011–2013.

The total sample comprised 170 journals, published by 54 academic publishers, with headquarters in 20 countries. Four publishers were responsible for publishing nearly half (49%) of the retrieved journals, namely Elsevier (36 journals), Wiley-Blackwell (22 journals), Springer (13 journals) and Nature Publishing Group (12 journals). Regarding country of origin, 95% of all journals were either based in the EU (81/170, 47 of which in the United Kingdom and 11 in the Netherlands) or the United States of America (USA) (80/170). The mean impact factor of the journals in this sample was 4.79 (Median = 3.63; Standard Deviation = 3.85). The median year of publication of first issue was 1987, with 75% of journals preceding 2002. Figure 2 represents the distribution of journals by field of research (of the three fields reviewed for this present study).

### 3.2. EXEMPLAR Score

**Figure 3:**
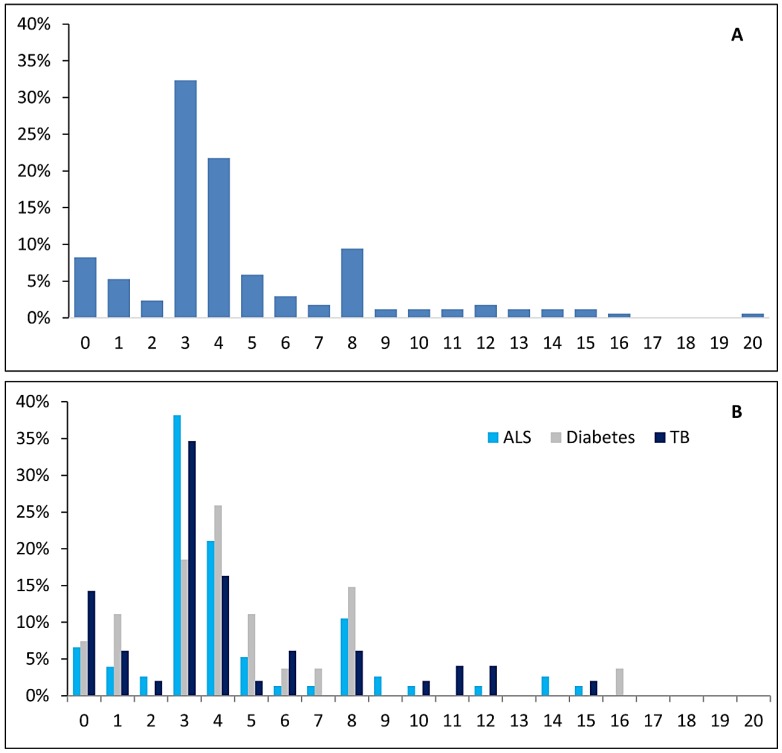
EXEMPLAR score distribution for the whole sample (Figure 3A; N = 170) and by field of research [Figure 3B, with 18 journals publishing studies in more than one field excluded; n(ALS) = 76; n(T1D) = 27; n(TB) = 49)]. The median score for the whole sample (N = 170) was 4 points, whereas the mean scores for ALS, T1D and TB were, respectively 4.45 (SD = 3.15), 4.52 (SD = 3.286) and 4.20 (SD = 3.54).

**Figure 4:**
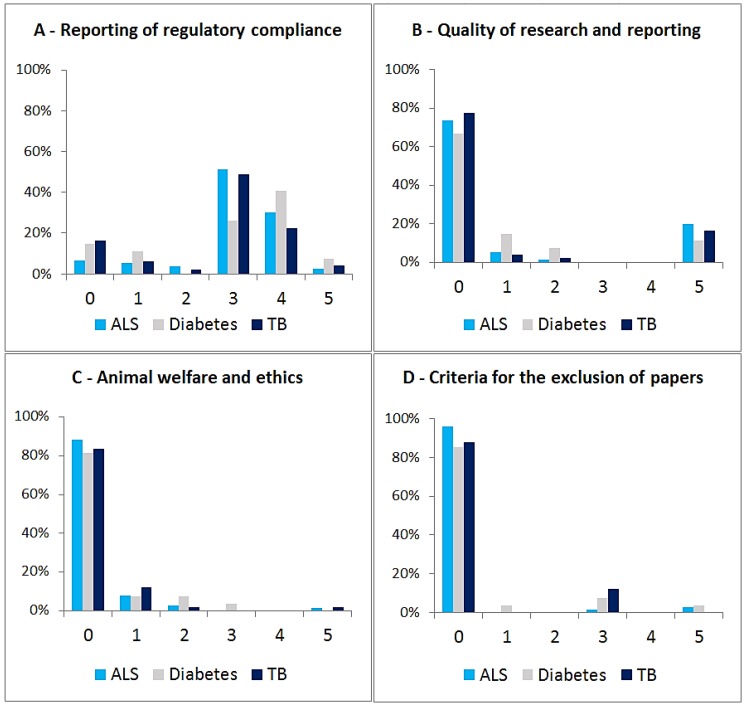
Scores for Categories **A**, **B**, **C** and **D**, for each. Journals publishing in more than one of the selected fields (n = 18) not shown [n(ALS) = 76; n(T1D) = 27; n(TB) = 49)].

No significant differences were found between the scores of the three sub-samples analyzed, the same happening for score distribution (Figure 3B). Results are hence presented for all journals pooled (*N* = 170, after removing duplicate entries from journals publishing in more than one field), unless stated otherwise. Category A (“reporting of regulatory compliance”) registered the highest non-nil score of all categories (92%), with 5% of journals being awarded the top score of five points. As for Category B (“Quality of research and reporting of results”), 72% had a nil score. However, 18% of journals were awarded the top score for this category, virtually all of these for referring to the ARRIVE guidelines. As for Category C (Ethical treatment of animals) and Category D (Criteria for the exclusion of papers), 86% and 91% of journals scored zero points, respectively (Figure 4). Overall, only 18% (31/170) of journals scored eight points or higher, with the two top-scoring journals being published by the Public Library of Science (*PLOS*), namely *PLOS One*, with a maximum score of 20 points, and *PLOS Genetics*, which scored 15 points (the two other PLOS journals in the sample scored five and 10 points).

### 3.3. Relationship between EXEMPLAR Score and Other Parameters

**Figure 5:**
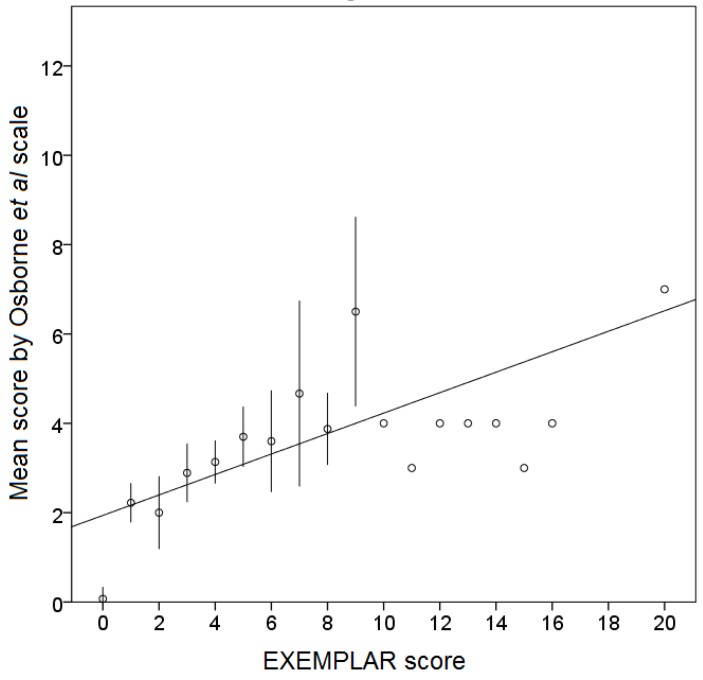
The mean “Osborne score” for journals with the same EXEMPLAR classification, with an overlaid best-fit regression line (R^2^ = 0.39). Error bars represent ± 1 standard deviation of the mean for “Osborne score”, for a confidence interval of 95%. No journal was classified with an EXEMPLAR score of 17, 18 or 19 points. Correlation was stronger (Pearson’s *r* = 0.736), for lower scoring journals (under 10 points in the EXEMPLAR scale, corresponding to 91% of the sample).

**Figure 6:**
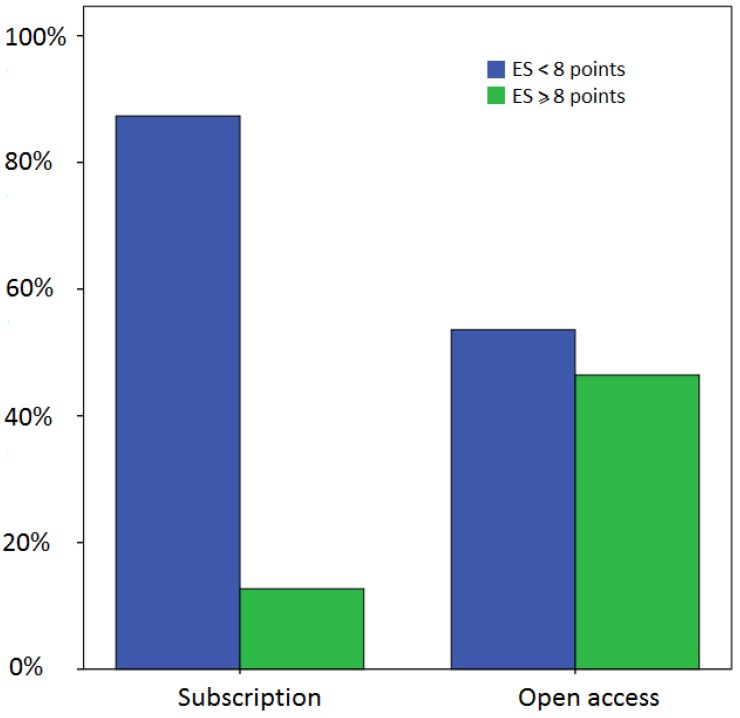
The proportion of open access journals with an ES ≥ 8 is significantly higher than that of subscription-based journals.

EXEMPLAR score and scores by the Osborne *et al.* scale (Figure 5) showed a positive correlation (Pearson’s r = 0.623, *p* < 0.001). The sample showed a mean “Osborne score” of 2.98 points with a standard deviation of 1.28. Less than 25% of journals’ scored more than three points (out of 12), and 8% of journals had a nil score by the Osborne *et al.* scale. Impact factor, country of origin, scientific category and first year of edition of scientific journals had no effect on the overall EXEMPLAR score. The model of publication (subscription *vs.* open access journals) was however shown to influence the overall EXEMPLAR score, as significantly more open access journals scored eight points or above (χ^2^(1) = 17.87, *p* < 0.001) than subscription based journals (Figure 6). Also, scores varied significantly between publishers (χ^2^(53) = 96.968, *p* = 0.001), as well as between journals from the same publisher.

## 4. Discussion and Conclusions

Our results suggest a more positive scenario than previous reviews of journal policies [27,28,29], as some level of concern is patent by most journals in our sample requiring a statement on regulatory compliance. However, the little progress found regarding in-house policies on the ethical treatment of animals is worrisome.

Both the EXEMPLAR and ‘Osborne *et al.*’s scoring of our sample paint the same overall scenario, and scores of the two classification schemes were, thus, not surprisingly, correlated. However, the EXEMPLAR allowed for a much higher differentiation between journals’ policies; for any given group of journals with the same ‘Osborne *et al.*’ score, several EXEMPLAR scores were often given; for instance, the 94 journals scoring three points by Osborne *et al.*’s scale were classified under ten different EXEMPLAR scores, ranging between one and 15 points. The latter included the journal with the second-most comprehensive and stringent policy on animal use, namely *PLOS Genetics*, with an *EXEMPLAR score* = ***15****:(5,5,5,0)*. Additionally, the top-scoring journal *PLOS One* [*EXEMPLAR score=* **20**:(*5,5,5,5*)] only received six points by the ‘Osborne *et al.*’ classification’. These discrepancies likely result from the EXEMPLAR covering some relevant items not contemplated by Osborne *et al.*’s scale, as well as from the different weight given to some items on the overall score. Also, we consider the EXEMPLAR to be more grounded on what in the current landscape can reasonably be expected from a journal with the highest standards, whereas some of the ‘Osborne *et al.*’ criteria could be considered more unrealistic (e.g., expecting journals to “demand that authors ‘improve on minimum standards set out in the relevant legislation”) or unnecessary (for instance, no journals demanded that “investigators and all personnel who handle and use animals are appropriately trained and qualified”, a likely consequence of journals assuming that only adequately trained professionals are granted licenses to work with animals).

Of all the parameters analyzed, only model of publication showed to significantly impact the overall EXEMPLAR score, which may be a consequence of open access journals being more freely accessible and hence more open to public scrutiny. This finding apparently contradicts a previous report [29] that accessibility to articles played no influence on the ethical stance of scientific journals on animal use. The latter, however, focused only on whether journals “required demonstrating adherence to any ethical guidelines”, a very superficial view of ethical standards that by the EXEMPLAR scale would be solely covered by Category A. The said study also proposed that older journals were more likely to require authors to provide a statement of regulatory compliance, a claim also challenged by our data. It has also been suggested that high impact factor journals may request less details on the animal experiments they publish [24]. At least judging from what is explicitly required by journals in their instructions for authors, we have not found any evidence that such a relationship could exist.

Most journals received one or more points for Category A, for regulatory compliance, in line with previous findings that statements of compliance with animal care guidelines and ethical approval of protocols have been on the rise and are now almost universally present in biomedical papers [30,31]. However, and considering the overall poor performance of journals in the other categories, this may imply that publishers transfer their responsibilities regarding the ethical treatment and use of animals to the institutions where the studies are carried out, or its regulators. This is particularly troublesome since the mere statement of compliance to animal care guidelines, or that a given project has been approved by regulators, do not necessarily mean that said project has gone through a thorough and balanced harm-benefit (*i.e.*, “ethical”) appraisal. Moreover, even if a project is indeed evaluated by a third party, principles and practice may vary greatly between different ethics committees (or equivalent), institutions or countries, as shown for instance by Plous and Herzog in 2001, who found an 80% disagreement between the decisions of different institutional animal care and use committee (IACUCs) over the same protocol [32].

From a regulatory point-of-view, project evaluation is often a “hybrid” between external (*i.e.*, legislation) and internal (by the scientific community) regulation of animal research, as it is usually a legal requirement, but on the other hand primarily carried out by scientists or others working in scientific institutions [33], which can lead to biases and conflicts of interests. Moreover, even if ethical appraisal is impartially carried out by knowledgeable and competent persons, this does not guarantee that the process is followed by any supervision or retrospective assessment, or that researchers going beyond the limits of their license are held accountable [34]. In fact, unacceptably severe studies (e.g., with death as an endpoint) may indeed be more prone to report any kind of regulatory compliance [30,31]. Despite these issues, since project approval does involve a third party to make a harm-benefit evaluation of the project, it was decided that it should have more weight on the overall score than the mere statement of compliance with norms and guidelines by authors, as these might be prompted by journal policy requirements, rather than having been in fact observed during the planning and execution of the experiments.

Although most journals had no explicit policies in place for the quality of reporting—covered by Category B—this category nonetheless obtained a higher mean score than the categories covering the ethical treatment of animals and the existence of criteria for the exclusion of papers. Indeed, 18% of journals were awarded the maximum score for this category, in virtually all of the cases for referring authors to the ARRIVE guidelines [23]. We opted to acknowledge journals for referring authors to reporting guidelines, even when compliance with said guidelines is not mandatory. The reasons behind our choice were, firstly, to be more realistic as regards to the current landscape, since only one journal in our sample—*PLOS Medicine*—made it mandatory to submit an ARRIVE checklist at the time of our analysis (*PLOS ONE* has, however, very recently changed its policies and now also requires submission of an ARRIVE checklist). Secondly, the actual enforcing of reporting guidelines relies on a clear understanding and communication between editors, reviewers and authors on this matter, which cannot be assessed by any analysis of the information made available in journals’ websites.

It is encouraging that journals are increasingly referring authors to reporting guidelines. However, the level of adherence is still very low, in line with recent reports that nearly half of the editors in veterinary journals are unaware of any guidelines for reporting of animal experiments [35]. The upholding of reporting standards by scientific journals is of central importance to allow an informed interpretation of published results, as well as to allow replication of the experiments, a cornerstone of scientific research. Also, having sufficient information on the experimental protocol and results available is of central importance for systematic reviews and meta-analyses, which provide evidence of treatment effects to inform clinical research [24]. Journals requiring detailed information on experimental design and statistics can also help improve the quality of research itself, as researchers wanting to publish their results in journals with high reporting standards must consider best practice in the project planning phase. Furthermore, currently available checklists listing key parameters of sound animal research (e.g., power analysis, random assignment of animals to treatment groups or blinding of observers [36]) or animal welfare (such as cage enrichments, pain alleviation or humane endpoints) can help researchers verify whether these are in place when planning and executing an experiment [37]. Attention to experimental design and adequate statistics can also allow for using only as many animals as scientifically necessary, hence following the 3Rs principle of *reduction* [36]. Additionally, improving journals’ adherence to reporting guidelines can help prevent the waste of animals’ lives and resources on irreproducible—on account of insufficient information in papers—or unreliable studies skewed by unaccounted biases and methodological errors [38,39,40,41].

The high number of nil-scoring journals for Category C—“Ethical treatment of animals”—is particularly worrisome, as the ethical [42], social [8] and legal [16] acceptability of animal experiments is typically grounded on a harm-benefit balance, which not only warrants animal use to be adequately justified, but also requires that experiments cause the least possible impact on animal welfare. It is also the category that focuses more on journals’ proactivity in promoting the ethical treatment of animals in research, thus becoming, as proposed by Bernard Rollin, true “guardians of the gates for animal welfare” [26]. The EXEMPLAR deliberately places a greater focus on refinement in this category, from the assumption that it is more reasonable to expect that editors and reviewers are willing—and have the needed expertise—to deal with the question of *how* studies submitted for publication were carried out than to judge if these should have rather been carried out by non-animal methods in the first place.

There are, however, limitations to what can be ascertained about how animals are treated from what researchers report, but editors and reviewers with reasons to question animal welfare standards should be able to ask authors the needed information to clarify this matter and determine if indeed animal suffering was excessive or unjustified. Furthermore, there is only so much that can be asked of reviewers, who for each paper they are asked to review have to carry out a specialized and unpaid work in a short period of time [43]. Also, the reviewers’ main task is usually to appraise manuscripts from a scientific point-of-view, being thus chosen for their expertise on a given field, rather than on ethics and animal welfare. These may hence lack the knowledge—and sometimes even the empathy—needed to adequately assess whether “all efforts were made to minimize suffering” (the standard, almost *cliché*, statement that can often be found in papers reporting unacceptably severe studies), or critically evaluate if the soundness and value of the study justify the cost borne by animals. Hence, editors’ duty must go beyond merely requiring a statement of compliance with local regulations [28,34,44].

The overwhelming majority of journals had no explicit statement on the exclusion of manuscripts for the unethical treatment of animals, covered by Category D. The absence of a statement demanding compliance with journal policies for manuscript acceptance does not necessarily imply that editors do not enforce them, but the fact that no measurable progress in the quality of reporting has been observed in journals endorsing the ARRIVE guidelines [45] raises the question of how effective journal policies are in promoting best practice in other aspects of animal research, as well, including those measured by the EXEMPLAR scale.

The conclusions that can be drawn from the data presented here have some limitations. Firstly, our sample was retrieved from a list of papers written in English, leaving out several scientific journals publishing in other languages such as Spanish, Portuguese, Mandarin or Russian, to name a few. Also, the EXEMPLAR scale was designed to classify explicitly stated journal policies, so scores may thus reflect “good intentions” rather than actual attitudes and practice by editors and reviewers. On the other hand, there might also be cases in which the opposite is true, as editors and reviewers who are sensitive to scientific and ethical issues on animal use may have their own set of criteria and apply them accordingly, regardless of explicitly stated journal policies. There may also be an inherent bias in our journal selection process, which aimed for high representativity of the selected fields (as the journals in the sample represent 75%, 73% and 72% of all ALS, T1D and TB papers published between 2011 and 2013, respectively) but on the other hand potentially limiting the extent to which these results can be extrapolated to the whole population of biomedical journals. There is, however, considerable diversity between the selected fields, and all major publishers were represented, as well as generalist journals, such as *PLOS ONE*, *Nature*, *PNAS* or the *EMBO journal*, among others. Furthermore, of all the journals retrieved from the original search results, only one was excluded for not publishing animal studies.

The disappointingly low scores found in our sample led us to consider the possibility that the EXEMPLAR could be setting unachievable standards. While a few high-scoring journals were found in our sample, we nevertheless analyzed a selection of five journals outside our original sample, reputed for their high standards regarding the ethical treatment of animals in research. These included two journals on laboratory animal science—*JAALAS* [EXEMPLAR =**16**:(2,5,5,4)] and *Laboratory Animals* [EXEMPLAR = **18**:(5,5,5,3)]—two others on animal welfare—*Animal Welfare* [EXEMPLAR= **15**:(4,5,3,3) and *Journal of Applied Animal Welfare* [EXEMPLAR=**12**:(2,2,5,3)]—and also MDPI’s *Animals* [EXEMPLAR = **15**:(5,5,5,3). These scores attest to the construct validity of the EXEMPLAR scale (*i.e.*, the degree to which it the scale measures what it intends to be measuring), as it allowed identifying quantitatively the (subjectively perceived) high standards of this selection of journals.

Overall, our data suggest there is still much to be done as regards the attention given by biomedical journals to the scientific and ethical issues underlying animal research. The main challenge will be finding how to make journal policies promote best practice, when most journals have failed [45] to ensure adherence to reasonable [44] principles of transparency and rigorous reporting, despite the set of guidelines and checklists now available for this purpose. A checklist for journal policies on animal welfare could hence also fail to have a meaningful impact in laboratory animals’ lives, because unlike reporting guidelines—that can be more broadly adopted—defining animal welfare requirements warrants a case-by-case approach, depending on the type of model used, choice of procedures and the scientific objectives. This may partially explain why compliance with regulations is a requirement for most journals, while having in-house policies in place on the ethical treatment of animals is not.

In conclusion, a re-evaluation of scientific journals’ current policies, as well as an improvement in the communication between editors and reviewers to implement such policies is of the essence [44]. A discussion of these results and of the standards upheld by the EXEMPLAR may be a good starting point.
